# Can cigarette smoking worsen the clinical course of cluster headache?

**DOI:** 10.1186/1129-2377-14-S1-P54

**Published:** 2013-02-21

**Authors:** I Tiraferri, F Righi, M Zappaterra, M Ciccarese, LA Pini, A Ferrari, MM Cainazzo

**Affiliations:** 1 Headache and Drug Abuse Interdepartmental Research Centre and Anti-smoking Centre University of Modena and Reggio Emilia, Italy

## Introduction

Up to 90% of cluster headache (CH) patients have a prolonged history of cigarette smoking prior to the headache onset. It has been suggested a genetic link between CH and nicotine addiction and, also, that toxic agents found in cigarette smoke have a direct effect on the hypothalamus, a pivotal area for the pathogenesis of CH. [1-3]

## Purpose

To explore the relationship between cigarette smoking and the clinical course of cluster headache.

## Methods

All outpatients with cluster headache, diagnosed according to the criteria of ICHD-II, who were, consecutively, seen from October 2010 to April 2012 at the Headache Centre, were subjected to a phone interview by means a specific standardized questionnaire (29 items), administered, always, by the same trained post-graduate medical doctor.

## Results

A total of 200 patients were surveyed (172 male, 28 female; mean age ± SD: 48.4 ± 12.7; male/female ratio: 6.1:1). One hundred and twenty patients were current smokers, 42 former smokers and 38 non-smokers. The age of onset of CH was 29.8 ±13.6 years. Among all smokers and former smokers those who started smoking before age of 18 years had an onset of cluster headache earlier than those who started smoking after age of 18 years (P < .01, Student’s t test). All patients with chronic cluster headache were currently smokers. The episodic form (89%) was more frequent than the chronic one (11%). Chronic CH patients smoked more cigarettes per day (P < .01, Student’s t test) and started smoking before (P < .01, Student’s t test) than patients with episodic CH (P = .001, Student’s t test). The length of the active phase of CH was tripled compared to non-smokers (weeks ± SD: 15.1 ± 17.6 vs. 5.7 ± 4.7; P < .001, Student’s t test).

## Conclusion

Our data showed that cigarette smoking is an aggravating factor for cluster headache, in particular for the lasting of the active phase.

## Competing interests

None.
